# Transient alteration of the vestibular calyceal junction and synapse in response to chronic ototoxic insult in rats

**DOI:** 10.1242/dmm.021436

**Published:** 2015-10-01

**Authors:** Lara Sedó-Cabezón, Paulina Jedynak, Pere Boadas-Vaello, Jordi Llorens

**Affiliations:** 1 Departament de Ciències Fisiològiques II, Universitat de Barcelona, 08907 L'Hospitalet de Llobregat, Catalonia, Spain; 2 Departament de Ciències Mèdiques, Facultat de Medicina, Universitat de Girona, 17071 Girona, Catalonia, Spain; 3 Institut d'Investigació Biomèdica de Bellvitge (IDIBELL), 08907 L'Hospitalet de Llobregat, Catalonia, Spain

**Keywords:** Ototoxicity, Vestibular system, Calyx endings, CASPR1, Calyceal junctions, Ribbon synapses, 3,3′-Iminodipropionitrile

## Abstract

Ototoxicity is known to cause permanent loss of vestibule function through degeneration of sensory hair cells (HCs). However, functional recovery has been reported during washout after chronic ototoxicity, although the mechanisms underlying this reversible dysfunction are unknown. Here, we study this question in rats chronically exposed to the ototoxic compound 3,3′-iminodipropionitrile (IDPN). Pronounced alterations in vestibular function appeared before significant loss of HCs or stereociliary coalescence became evident by ultrastructural analyses. This early dysfunction was fully reversible if the exposure was terminated promptly. In cristae and utricles, the distinct junctions formed between type I HCs (HCI) and calyx endings were completely dismantled at these early stages of reversible dysfunction, and completely rebuilt during washout. Immunohistochemical observations revealed loss and recovery of the junction proteins CASPR1 and tenascin-C and RT-PCR indicated that their loss was not due to decreased gene expression. KCNQ4 was mislocalized during intoxication and recovered control-like localization after washout. At early stages of the intoxication, the calyces could be classified as showing intact or lost junctions, indicating that calyceal junction dismantlement is triggered on a calyx-by-calyx basis. Chronic toxicity also altered the presence of ribeye, PSD-95 and GluA2 puncta in the calyces. These synaptic alterations varied between the two types of calyx endings (formed by calyx-only or dimorphic afferents) and some persisted at the end of the washout period. The present data reveal new forms of plasticity of the calyx endings in adult mammals, including a robust capacity for rebuilding the calyceal junction. These findings contribute to a better understanding of the phenomena involved in progressive vestibular dysfunction and its potential recovery during and after ototoxic exposure.

## Abstract (precis)

**Summary:** New forms of damage and repair have been identified in the vestibular sensory epithelium using a rat model of chronic ototoxicity and recovery that causes reversible vestibular dysfunction.

## INTRODUCTION

Impaired function of the vestibular system causes vertigo, loss of balance and loss of gaze fixation during movement, often accompanied by dizziness and nausea. One cause of vestibular dysfunction is inner ear damage following exposure to ototoxic chemicals. These include therapeutic drugs, such as aminoglycoside antibiotics and the chemotherapeutic agent cisplatin, as well as a number of workplace chemicals and environmental pollutants (Forge and Schacht, 2000; Sergi et al., 2003; Hodgkinson and Prasher, 2006; Schacht et al., 2012; Saldaña-Ruíz et al., 2012a,b; Sedó-Cabezón et al., 2014). A well-established result of ototoxic insult is the loss of the sensory hair cells (HCs) responsible for mechanotransduction in both the vestibular and auditory systems. In fish, avian and amphibian species, the sensory epithelium produces new HCs to replace lost ones, synapses are recovered and tissue repair results in functional recovery (reviewed by Rubel et al., 2013), although the application of cisplatin may block regeneration (Slattery and Warchol, 2010). By contrast, mammalian vestibular HCs do not readily regenerate (Llorens et al., 1993; Llorens and Demêmes, 1994). Although HC regeneration capacity persists to a limited extent in some mammalian species (Forge et al., 1993; Warchol et al., 1993), it does not provide significant functional recovery (Forge and Schacht, 2000; Groves, 2010; Rubel et al., 2013). Studies in experimental animals contrast with observations in humans after discontinuation of chronic ototoxic exposure. Thus, patients halting aminoglycoside use after diagnosis of ototoxicity experience variable outcomes, from full persistence of the symptoms through partial recovery to complete recovery (Black et al., 2001, 2004). Although CNS compensation may partly account for behavioral recovery (McCall and Yates, 2011), very little is known about the cellular and molecular mechanisms involved in chronic vestibular damage and possible repair, and their relevance to the observed functional loss and possible recovery. Human data are very rare (Tsuji et al., 2000) and provide only limited information on major pathological alterations.

Previously, understanding of the physiopathology of chronic vestibular toxicity was limited, at least in part, by the reduced flexibility of the available rat and mouse models of chronic aminoglycoside and cisplatin ototoxicity. Owing to the relative resiliency of these species to the HC toxicity compared with other toxicities of these drugs, it is difficult to study specific aspects of the ototoxicity as dosing and exposure times cannot be altered without encountering either excessive systemic toxicity or insufficient ototoxicity (Wu et al., 2001; Murillo-Cuesta et al., 2010; Oesterle et al., 2008; Taylor et al., 2008; Li et al., 2011). An alternative model is offered by ototoxic nitriles, including 3,3′-iminodipropionitrile (IDPN), which are much easier to use in rats and mice. The ototoxic properties of nitriles are very similar to those of aminoglycoside antibiotics. They affect both the auditory and the vestibular system (Llorens et al., 1993; Crofton et al., 1994; Balbuena and Llorens, 2001; Gagnaire et al., 2001; Soler-Martín et al., 2007) and show selectivity for HCs as the primary target (Llorens and Demêmes, 1994). The selectivity of aminoglycosides and cisplatin for HCs has been demonstrated in multiple mammalian and non-mammalian species, and the susceptibility of very diverse species has also been demonstrated for the nitriles (Soler-Martín et al., 2007). Like the antibiotics, nitriles also show the characteristic intra-epithelial and inter-epithelial differences in susceptibility: hair cell loss progresses in a basal to apical order in the cochlea; in a central to peripheral order within each vestibular epithelia; and in a crista to utricle to saccule order across the vestibular epithelia (Llorens et al., 1993; Llorens and Demêmes, 1994; Balbuena and Llorens, 2001, 2003; Soler-Martín et al., 2007). Humans can be exposed to natural (Tanii et al., 2004) and industrial (Saldaña-Ruíz et al., 2012a) ototoxic nitriles, but clinical evidence of their ototoxicity is not available. Also, the mechanisms involved in their ototoxicity remain unexplored, so it is feasible that many differences exist within those mechanisms identified for aminoglycosides and cisplatin (Schacht et al., 2012). Nevertheless, the experimental data available clearly indicate that nitriles exert a selective toxic effect on HCs, and therefore their study may reveal phenomena potentially relevant to a variety of ototoxicity.

**TRANSLATIONAL IMPACT**

**Clinical issue**

Vestibular dysfunction might occur as a consequence of exposure to ototoxic chemicals, which are known to cause loss of the vestibular sensory hair cells (HCs). However, other ototoxic effects remain much less understood. Scarce information is available on the cellular and molecular basis of the progression of vestibular dysfunction during chronic ototoxicity and its potential reversibility. The extent of functional recovery is highly variable among patients, and the basis of this variability is not known. Progress in this area is hampered at least in part by the limited flexibility of the currently available rodent models of ototoxicity that are based on the use of the clinically relevant compounds cisplatin and aminoglycosides.

**Results**

In this study, the authors used 3,3′-iminodipropionitrile (IDPN) to study the progression of vestibular dysfunction and its reversibility during and after chronic ototoxic exposure in rats. Although significant exposure to IDPN does not occur in humans, its ototoxic properties are very similar to those of the aminoglycosides. Using this reliable model, the authors observed that severe vestibular dysfunction appears before major damage to the HCs occurs, and that this early dysfunction is reversible if the exposure is halted promptly. The authors identified one prominent early effect in chronic toxicity, i.e. the dismantlement of the calyceal junctions, a specialized adhesion complex formed between the amphora-shaped type I HCs and the post-synaptic calyx nerve terminals. This ultrastructural evidence was corroborated by immunohistochemical data demonstrating major reductions in the content of the junction proteins CASPR1 and tenascin-C and redistribution of the voltage-gated potassium channel KCNQ4 in the epithelia. Chronic ototoxicity also caused a decrease in a number of synaptic components (including ribeye, GluA2 and PSD-95). Data obtained after IDPN washout demonstrated a robust capacity for calyx repair and calyceal junction rebuilding, and indicated that synaptic elements are differentially regulated across these processes.

**Implications and future directions**

This model of chronic ototoxicity and washout in rats has revealed new forms of damage and repair in the vestibular sensory epithelium, providing a hint on the events that, besides HC degeneration, might occur in the labyrinth of humans exposed to ototoxic agents. Understanding the cellular and molecular basis of ototoxic damage and repair might provide candidate targets for halting damage progression and promoting repair of the system. Some of these could also be relevant for other vestibular diseases or for age-related sensory decline. At present, therapeutic options for vestibular diseases are almost nonexistent.

One major question in vestibular toxicity is the involvement of afferent terminals and their potential for repair. Reversible afferent damage could explain the recovery of lost function. Although the major ototoxic compounds are usually regarded as being selectively toxic to HCs (Schacht et al., 2012), damage to the afferents has also been observed to occur *in vivo* (Sera et al., 1987; Hirvonen et al., 2005). There are three types of afferents forming two types of endings onto two different types of HCs. Calyx endings envelope the amphora-shaped type I HCs (HCIs), and button endings contact the more cylindrical type II HCs (HCIIs). Afferents may form only calyces (calyx-only, expressing calretinin), only buttons (button-only) or both types of endings (dimorphic afferents, calretinin negative). The common neurotransmitter is glutamate and the afferents are susceptible to excitotoxic damage (Raymond et al., 1988). It has been demonstrated that calyx endings in rats can be repaired after being acutely damaged by intratympanic exposure to the non-NMDA glutamate receptor agonist kainic acid (Brugeaud et al., 2007; Dyhrfjeld-Johnsen et al., 2013). However, data from local aminoglycoside application to chinchilla suggest that afferents may show persistent alterations after ototoxicity (Hirvonen et al., 2005). In the chronic systemic ototoxicity model offered by drinking water exposure to IDPN in rats, detachment, retraction and fragmentation of the calyx endings precede the later HC demise that occurs by extrusion of the cells from the sensory epithelium to the endolymphatic cavities (Seoane et al., 2001a,b). In this model, rats showed no significant recovery of vestibular dysfunction after termination of exposure at 13 weeks, in accordance with the extensive HC loss observed in the sensory epithelia (Llorens and Rodríguez-Farré, 1997). By contrast, in some unpublished experiments, we observed complete functional recovery if the exposure was terminated as soon as overt dysfunction was observed. We hypothesized that this model could be useful to identify the molecular basis for these effects and their potential reversibility.

A remarkable feature of the vestibular epithelium is the electrodense junction formed by the membrane on the inner side of the calyx and the lower two-thirds of the plasma membrane of the HCI. This calyceal junction, prominent at the transmission electron microscopy (TEM) level, is related to the invertebrate septate junction (Sousa et al., 2009) and is similar to the paranodal junctions formed by the axons and loops of myelinating cells (Einheber et al., 1997). The calyceal junction area has been defined as a functional microdomain (domain 1) containing many specific proteins not present in other parts (domains 2, 3 and 4) of the calyx nerve terminal (Lysakowski et al., 2011). A major component is CASPR1 (contactin-associated protein), which is a homolog of the *Drosophila* septate junction protein neurexin IV, and it has been identified as a key component of mammalian paranodal junctions (Einheber et al., 1997). Heterodimers of CASPR1 and contactin-1 form the axonal side of the paranodal junction, and *Caspr1*-null mice fail to form the calyceal junctions (Sousa et al., 2009). The extracellular matrix of the calyceal junction area has been shown to contain tenascin-C (Swartz and Santi, 1999; Lysakowski et al., 2011), a homohexameric protein also known to interact with contactin-1 (Falk et al., 2002). The voltage-gated potassium channel KCNQ4 also colocalizes with CASPR1 in the calyceal junction area in control vestibular epithelia (Sousa et al., 2009; Lysakowski et al., 2011), and its distribution is altered in *Caspr1*-null mice (Sousa et al., 2009). Although the calyceal junction defines a large area of proximity and relationships between the calyx ending and the HCI, the synaptic transmission machinery occupies smaller discrete zones (Lysakowski et al., 2011; Sadeghi et al., 2014). The presynaptic active zones contain ribbons, characterized by the ribeye/CtBP2 core protein, whereas the calyces contain post-synaptic densities rich in PSD-95, where the glutamate AMPA receptors are clustered. In the cochlea, pairing of ribeye/CtBP2 immunofluorescent puncta and GluA2/3 puncta has been used to estimate the number of active synapses, and decreased numbers have been interpreted as an indication of synaptic uncoupling (Sergeyenko et al., 2013; Moser et al., 2013). In the vestibular calyx synapse, poor juxtaposition of presynaptic ribeye and postsynaptic GluA2/3 has recently been demonstrated to be a physiological feature, and this distribution supports the hypothesis that neurotransmission in this synapse may rely considerably on glutamate spillover (Sadeghi et al., 2014).

Here, we present evidence that dismantlement of the calyceal junction is an early event in afferent damage during chronic IDPN ototoxicity and that extensive repair of the junctions is possible upon termination of the insult. Early changes also include alterations in the synaptic transmission machinery, repair of which does not match that of the calyceal junctions. These data have been obtained in an ototoxicity and washout model causing significant vestibular dysfunction and recovery.

## RESULTS

### Chronic IDPN exposure causes progressive vestibular dysfunction that may revert if exposure is halted promptly

**Fig. 1.**
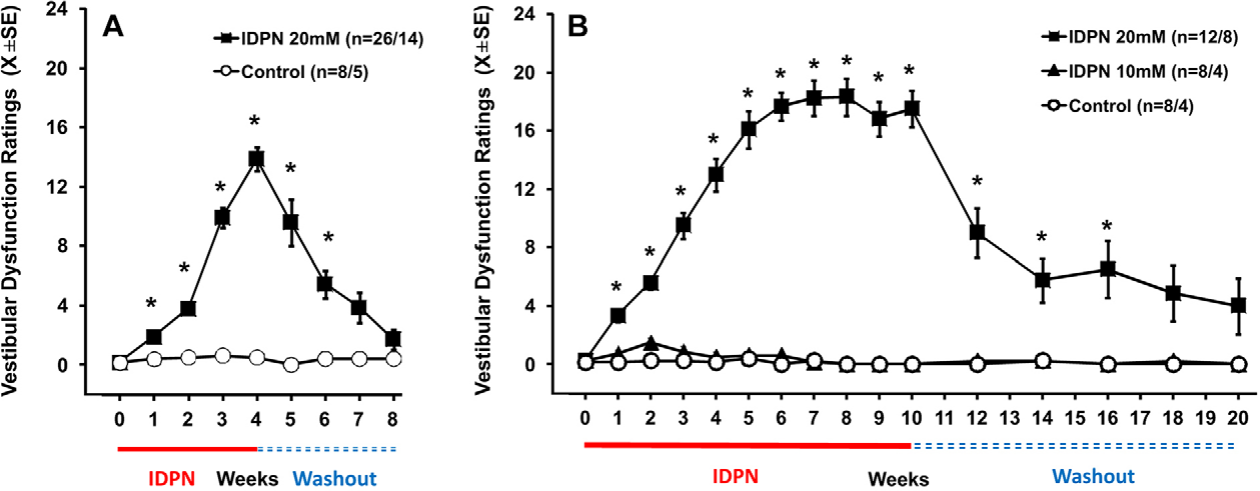
**Functional effects of chronic ototoxic exposure and washout.** (A,B) Rats were administered 0 (control), 10 or 20 mM IDPN in their drinking water for 4 (A) or 10 (B) weeks. Data are ratings for vestibular dysfunction from a battery of behavioral tests. The animals were assessed weekly during exposure and at one- or two-week intervals during a washout period of similar length. The higher number of animals per group corresponds to the total number of animals during the exposure period; the lower number to the animals remaining in the washout period. *P<0.05.

To study the processes involved in vestibular damage and repair during chronic IDPN ototoxicity and subsequent recovery, we exposed male Long-Evans rats to IDPN via drinking water for several weeks and assessed them at the end of exposure or after a washout period. In accordance with previous data (Llorens and Rodríguez-Farré, 1997), rats allowed free access to 10 or 20 mM IDPN received fairly stable doses, estimated on cage group data to be 0.43±0.03 and 0.69±0.08 mmol/kg/day (X±s.d.), respectively. Exposure to the higher of these concentrations for 13 weeks has been demonstrated to cause loss of most HCs in cristae and utricles (Seoane et al., 2001a) and persistent vestibular dysfunction (Llorens and Rodríguez-Farré, 1997). In the present study, rats exposed to 20 mM IDPN in drinking water for 4 weeks attained substantial but not maximal (6-18 on a scale of 0-24) vestibular dysfunction ratings (VDRs; Fig. 1A). Discontinuation of exposure at that time point resulted in a progressive reduction in dysfunction ratings, and after a washout period of 4 weeks only one out of the 14 animals displayed a substantial rating of ten, whereas the remaining 13 animals showed ratings in the control range (0 to 2). Weekly group mean comparisons of the VDR of the animals allowed to survive for 8 weeks resulted in significant week (*F*[8,10]=16.2, *P*<0.001), treatment (*F*[1,17]=26.1, *P*<0.001), and week×treatment interaction (*F*[8,10]=12.5, *P*<0.001) effects, reflecting the higher mean VDR of the treated animals than of control animals. To study longer exposure periods likely to cause deeper damage at the same concentration, additional groups of rats were exposed to 0 or 20 mM IDPN for 6 weeks (VDR mean data not shown) or to 0, 10 or 20 mM IDPN for 10 weeks (Fig. 1B). The rats exposed for 10 weeks to 20 mM IDPN showed higher dysfunction scores than rats exposed for 4 weeks, and two out of four animals in this group failed to show complete recovery after a washout period of 10 weeks. By contrast, animals exposed to 10 mM showed no overt behavioral dysfunction after up to 10 weeks of dosing. Analysis of the VDR of animals exposed for 10 weeks and allowed to survive for 10 additional weeks indicated significant week (*F*[3,3]=12.1, *P*<0.001), treatment (*F*[1,13]=42.0, *P*<0.001), and week×treatment interaction (*F*[6,6]=15.9, *P*<0.001) effects. Significantly increased mean scores (*P*<0.05) were recorded for the 20 mM animals from week 1 to 16 of the experiment. Among the rats allowed to recover after termination of exposure in the three experiments, all eight with VDR≤17 at the end of the exposure period showed complete behavioral recovery during the washout period (final scores in the 0 to 3 range) whereas five out of the 11 rats (45%) with scores ≥18 showed incomplete recovery (final scores >9).

Taken together, these data indicate that good recovery can occur even in animals showing pronounced vestibular dysfunction, but that more pronounced loss and longer exposure increase the risk of persistent dysfunction. They also demonstrate that animals with similar loss of function as assessed by behavioral evaluation may show different degrees of recovery. This exposure model is thus adequate for studying the cellular and molecular basis of the functional loss and recovery that occurs in chronic ototoxicity.

### Vestibular dysfunction appears before coalescence of stereociliary bundles and HC loss

**Fig. 2.**
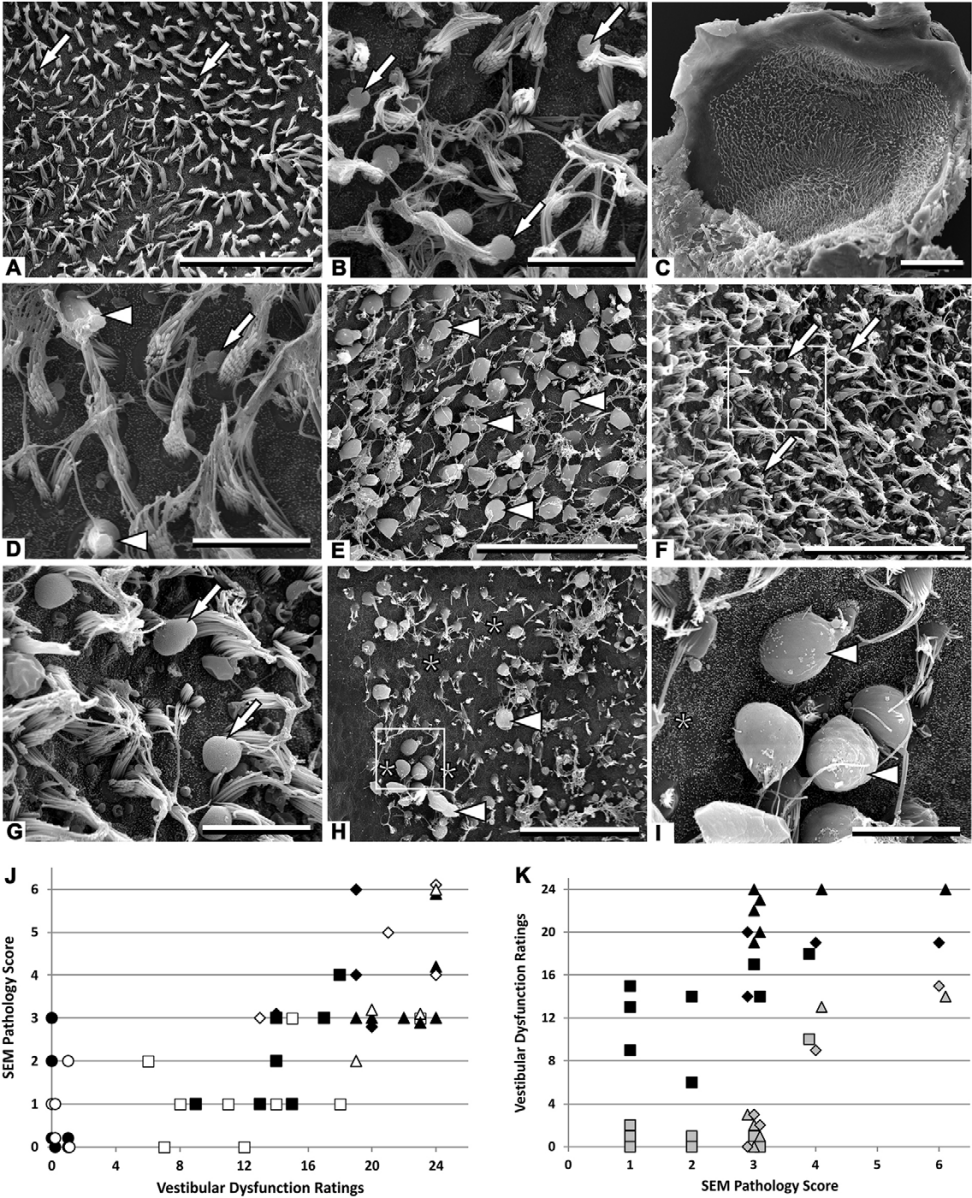
**Effects of chronic ototoxic exposure (20 mM IDPN in drinking water) and washout on the vestibular sensory epithelium as observed in surface preparations of utricles examined by SEM.** (A) Control rat. Only a few small blebs behind stereociliary bundles (arrows) are noted as possible pathological feature in this control sample. (B) Rat exposed for 4 weeks, and with a vestibular dysfunction rating (VDR) of 14. Blebs behind stereociliary bundles (arrows) were the single noticeable pathological feature. (C) Rat exposed for 4 weeks, with a VDR of 23, displaying a control-like appearance at low magnifications. (D) Higher magnification view of the utricle in C revealing only a few pathological features. In addition to blebs (arrow), a modest proportion of stereociliary bundles showed coalescence (arrowheads). (E) Rat exposed for 10 weeks, with a VDR of 24. Note that the majority of the HCs (arrowheads) are extruding into the endolymphatic cavity through the stereociliary bundle. (F) Rat exposed for 6 weeks examined after a washout period of 6 weeks. This animal showed a good recovery: VDR decreased from 24 at the end of the exposure to 3 at the time of histological examination. Blebs (arrows) were abundant and large, but little evidence of HC extrusion or loss was present. (G) Higher magnification view of the boxed area in F. Arrows indicate blebs. (H) Rat exposed for 6 weeks examined after a washout period of 6 weeks. This animal failed to recover well: VDR only varied from 24 at the end of the exposure to 14 at the time of histological examination. Extruding HCs (arrowheads) were abundant and areas containing only supporting cells (asterisks) denoted the loss of HCs. (I) Higher magnification view of the boxed area in H. Arrowheads indicate extruding HCs; asterisk indicates areas containing only supporting cells. (J) Relationship between VDRs and SEM Pathology Scores in control animals (circles) and in animals treated with 20 mM IDPN for 4 (squares), 6 (triangles) or 10 (rhombus) weeks; animals were examined at the end of the exposure (open symbols) or after a washout period (filled symbols). (K) Relationship between SEM Pathology Scores and VDR recovery in animals exposed to 20 mM IDPN and examined after a washout period. Two symbols are included for each animal, showing the VDR at the end of the exposure period (black), and at the end of the washout period (gray). Symbol shape as in G. Scale bars: 50 µm in A,E,F and H; 10 µm in B,D,G and I; 100 µm in C.

To identify the stages of damage that are amenable to repair and may underlie functional recovery, we examined the ultrastructural features of the vestibular cristae and utricles. Fig. 2 illustrates the results of the scanning electron microscopy (SEM) evaluation of the surface preparations of the utricles; findings in cristae were similar. Control epithelia from this series of experiments (*n*=11) included no pathological features (*n*=6, SEM pathology score=0; see Materials and Methods section for a description of the scores); few and small surface blebs appearing behind the stereociliary bundles (*n*=2, SEM pathology score=1; Fig. 2A); larger or more abundant blebs (*n*=2, SEM pathology score=2); and presence of blebs and very few damaged stereociliary bundles (*n*=1, SEM pathology score=3). The surface blebs are known to form quickly in case of HC stress (Shi et al., 2005; Goodyear et al., 2008) and are a common preparation artifact (Seoane et al., 2001b). The vestibular epithelia of animals with dysfunction scores ≤17 at the end of the exposure period consistently showed no evidence of major stereocilium abnormalities (SEM pathology scores in the 0 to 3 range; Fig. 2B). This included seven out of eight animals exposed for 4 weeks but also one out of four animals exposed for 10 weeks. Control-like stereociliary bundles were also recorded when these moderately affected animals were examined after the washout period (not shown).

In animals with scores ≥18, different conditions were recorded. In some cases, the appearance was almost control-like. Although blebs behind stereociliary bundles were more abundant on average than in control specimens, few coalescent bundles were observed (SEM pathology score=3; Fig. 2C,D). In other cases, the epithelium showed abundant presence of abnormal stereociliary bundles with fused stereocilia and HC extrusion (SEM pathology scores in the 4 to 6 range; Fig. 2E). When the animals were examined after the washout period, diverse outcomes were again observed and related to vestibular dysfunction. Thus, animals that had shown behavioral recovery displayed no or only mild pathological alterations of the hair bundles. In some cases, blebs behind the bundles were abundant but evidence of HC loss and stereociliary coalescence was scarce (SEM pathology score=3; Fig. 2F,G). By contrast, animals that had failed to recover control-like behavior displayed areas containing only supporting cell surfaces, denoting HC loss, as well as abundant coalescent stereociliary bundles and extruding HCs (SEM pathology score=6; Fig. 2H,I).

The relationship between the vestibular dysfunction of the animals at the end of the exposure period and the SEM pathology scores is summarized in Fig. 2J. Fig. 2K shows the relationship between the SEM pathology scores and the VDRs at both the end of the exposure period and after recovery. Taken together, these data demonstrated that significant vestibular dysfunction occurs during chronic ototoxic stress before bundle coalescence spreads out, and that this functional loss is amenable to substantial recovery. Our observations also indicate that stereociliary coalescence and HC extrusion are associated with a poorer prognosis for functional recovery, suggesting that stereociliary coalescence may be the point of no return for adult rat HCs.

### Disaggregation of the calyceal junctions and calyx ending fragmentation are reversible events associated with chronic IDPN ototoxicity and washout

**Fig. 3.**
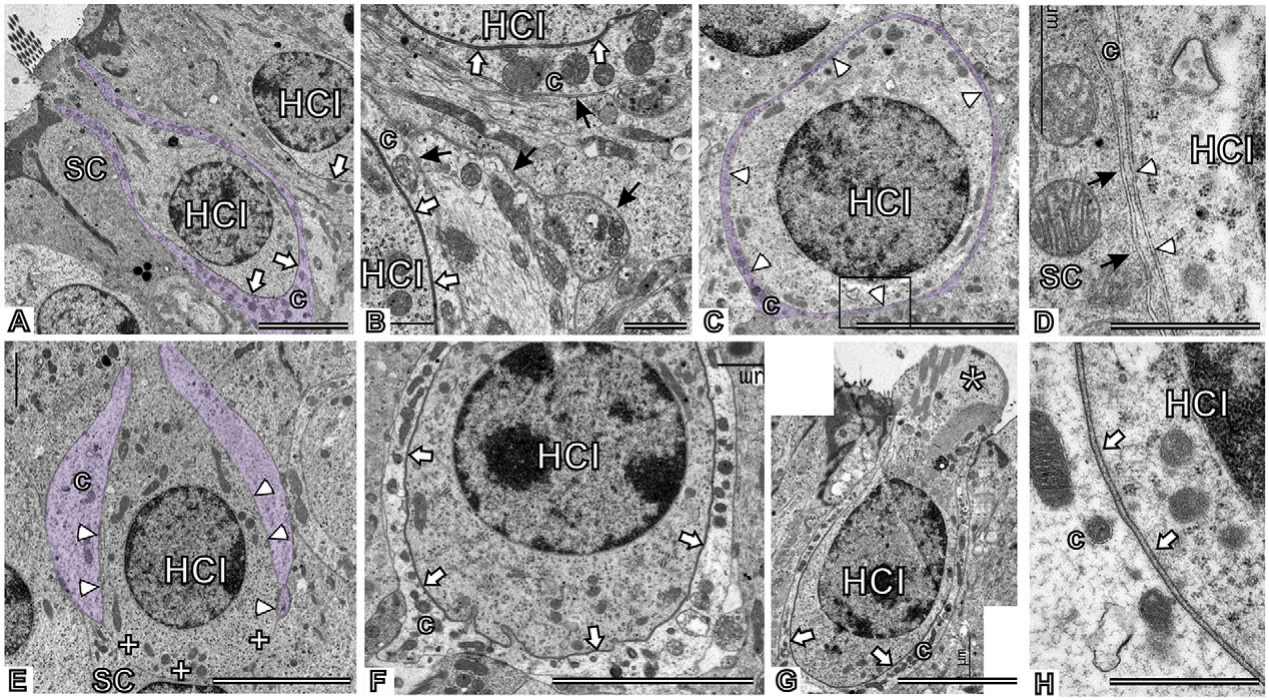
**Effects of chronic ototoxic exposure (20 mM IDPN in drinking water) and washout on the vestibular sensory epithelium as observed by TEM.** (A) Control crista. A calyx ending (c) is highlighted by violet shading to show its normal shape, fully enveloping the HCIs up to the neck region. A calyceal junction (white arrows) is observed at the contact between the calyx ending and the HCI. (B) Higher magnification of a control crista. Note the difference in electron density between the calyceal junction membranes (white arrows) and other plasma membranes in the tissue (black arrows). (C-E) Cristae from animals exposed to IDPN for 4 weeks. Arrowheads point to calyx-HCI contact areas that lack calyceal junctions. D shows a higher magnification of the boxed area in C: note the similarity in the electron density of the membranes at the contact between the HCI and the calyx (arrowheads) with those at the neighboring contact between the calyx and the supporting cell (SC; black arrows). A fragmented calyx is shown in E; the calyx, shaded in violet, does not cover the basal region of the cell, marked with plus signs. Arrowheads indicate contact areas that lack calyceal junction features. This image is from the same animal shown in Fig. 2C and 2D. (F-H) Cristae from animals exposed to IDPN for 4 weeks and examined after a washout period of 4 weeks. Calyceal junctions (white arrows) are present. Note the apical damage (asterisk) to the cell in G; its calyceal junction, shown at higher magnification in H, had control-like features. Scale bars: 5 µm in A,C,E,F; 1 µm in B,D,H.

To reveal further ultrastructural features of the ototoxic insult and subsequent recovery, besides the stereociliary effects, cristae were examined by TEM. In control epithelia (*n*=3; Fig. 3A,B), the ultrastructural features conformed to descriptions in the literature of normal cristae (Wersäll and Bagger-Sjöbäck, 1974; Lysakowski and Goldberg, 1997; Seoane et al., 2001a; Sousa et al., 2009). In our control specimens, the calyx units were not swollen and calyceal junctions were consistently recognized in all calyceal contacts. In animals exposed to 20 mM IDPN for 4 weeks (*n*=5, VDR=11, 14, 15, 18 and 23), calyceal junctions were conspicuously absent (Fig. 3C-E). Among these animals, only the one with the lowest dysfunction rating showed HCI-afferent contacts with fragmented remainders of calyceal junctions, whereas their complete absence was recorded in the other four rats. In the two animals displaying the greatest loss of vestibular function, we found several calyx units that were fragmented; thus, regions of the basolateral membrane of the HCIs that are normally covered by the calyx ending were not (Fig. 3E). A remarkable observation was the absence of overt swelling of the calyces, including those with intact overall morphology but lost junctions (Fig. 3C) and those additionally showing evident fragmentation (Fig. 3E). This observation suggests that calyx detachment and retraction may not be a consequence of previous swelling.

Animals examined 4 weeks after the end of the 4-week period of exposure to 20 mM IDPN (*n*=4) displayed extensive repair of the calyx endings and calyceal junctions (Fig. 3F-H). This included the three rats showing full behavioral recovery (VDR=14, 15 and 17 at the end of the exposure; and 0, 2 and 1 after the washout period), but also the animal with incomplete behavioral recovery (VDR=18 at the end of the exposure; and 10 after the washout period). One striking observation in this last animal was the presence of HCIs with cytoplasm extrusion between the stereocilia but control-like calyceal junctions (Fig. 3G,H). Because calyceal junctions were almost completely dismantled at the end of the exposure in these animals, this observation indicates that the junctions can be rebuilt in HCI suffering severe apical abnormalities.

**Fig. 4.**
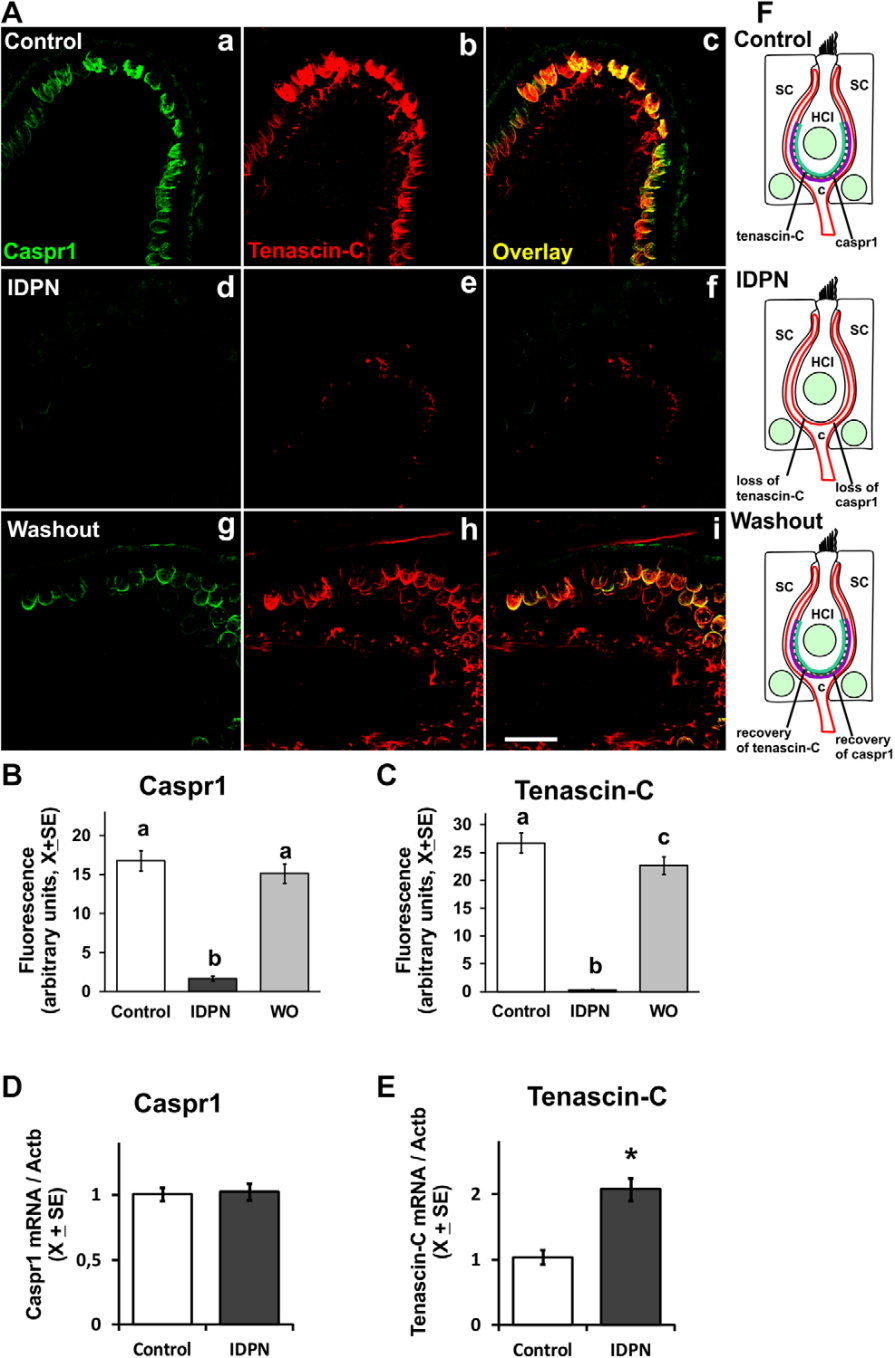
**Effects of chronic ototoxic exposure (20 mM IDPN in drinking water for 4 weeks) and washout on the expression of CASPR1 and tenascin-C in the vestibular periphery of the adult rat.** (A) Representative images of the sensory epithelia showing cristae immunolabeled with the K65/35 anti-CASPR1 antibody (green) and the AB19013 anti-tenascin antibody (red). In the overlay figures, yellow indicates overlapping green and red signals at this level of resolution. (a-c) Control; note the cup-shaped labeling of both proteins corresponding to their known localization in the inner faces of the calyx membranes (CASPR1) and the adjacent extracellular clefts (tenascin-C) of the calyceal junction area. (d-f) After exposure to IDPN for four weeks, labeling of both proteins is dramatically reduced. (g-i) After a 4-week washout period, extensive recovery is observed in the labeling. Scale bar: 25 µm. (B,C) Quantitative comparison of immunofluorescence intensity among calyx shapes between control, treated and washout animals (*n*=30 cells from 3 animals per group). a,b,c: groups not sharing a letter are significantly different, *P*<0.05, Duncan's test after significant ANOVA. (D,E) RT-PCR of mRNA from vestibular ganglia displays increased expression of tenascin-C but unchanged *Caspr1* (*n*=7-8 per group, *P<0.05). (F) Schematic summarizing the behavior of CASPR1 in the inner calyceal membrane and tenascin-C in the extracellular matrix during IDPN exposure and washout. HCI, Type I hair cell; SC, supporting cell; c, calyx ending.

To characterize further the dynamics of the afferent damage and repair processes, we used fluorescence immunohistochemistry to compare control animals with both animals showing intermediate dysfunction ratings (12 to 17) after 4 weeks of 20 mM IDPN and washout animals showing full behavioral recovery from a similar dysfunction after a period of 4 weeks. We focused on the expression of proteins previously identified at the calyceal junction area within the HCI-calyx ending contact. In exposed animals, we observed a marked reduction in CASPR1 immunoreactivity, but this label was clearly recovered after the 4-week washout period (Fig. 4A). Quantitative comparisons of the CASPR1 label indicated significant group differences (*F*[2,87]=59.45, *P*<0.001; *n*=30 cells from three animals per group) with clear loss after exposure and full recovery after the washout (Fig. 4B). These changes in CASPR1 matched the predictions made on the observed loss and recovery of the calyceal junctions by TEM. In accordance with previous observations (Seoane et al., 2003), we also recorded a depletion of neurofilaments in the afferent terminals of the IDPN-exposed animals (data not shown). Similarly to CASPR1, a marked loss of tenascin-C immunoreactivity was observed in the animals exposed to 20 mM IDPN for 4 weeks, with significant recovery observed at the end of the washout period (Fig. 4A). Quantitative analysis demonstrated a robust effect (*F*[2,87]=102.9, *P*<0.001; *n*=30 cells from three animals per group) with a dramatic loss and full recovery (Fig. 4C). Thus, CASPR1 and tenascin-C exhibit similar behavior during dismantlement and repair of the calyceal junction (Fig. 4F).

We also used RT-PCR to evaluate whether the loss of the calyceal junctions was explained by decreased mRNA expression of CASPR1, tenascin-C or contactin-1. The data obtained demonstrated no change in CASPR1 (Fig. 4D) or contactin-1 (not shown) expression in the vestibular ganglia. The expression of tenascin-C was not modified in the vestibular epithelia (not shown), but was significantly increased in the ganglia (Fig. 4E; *P*<0.001). Therefore, the loss of the calyceal junctions is not due to decreased gene expression of these major constitutive proteins; on the contrary, an increase was in fact recorded for tenascin-C in the ganglia, perhaps as a result of a compensatory response.

**Fig. 5.**
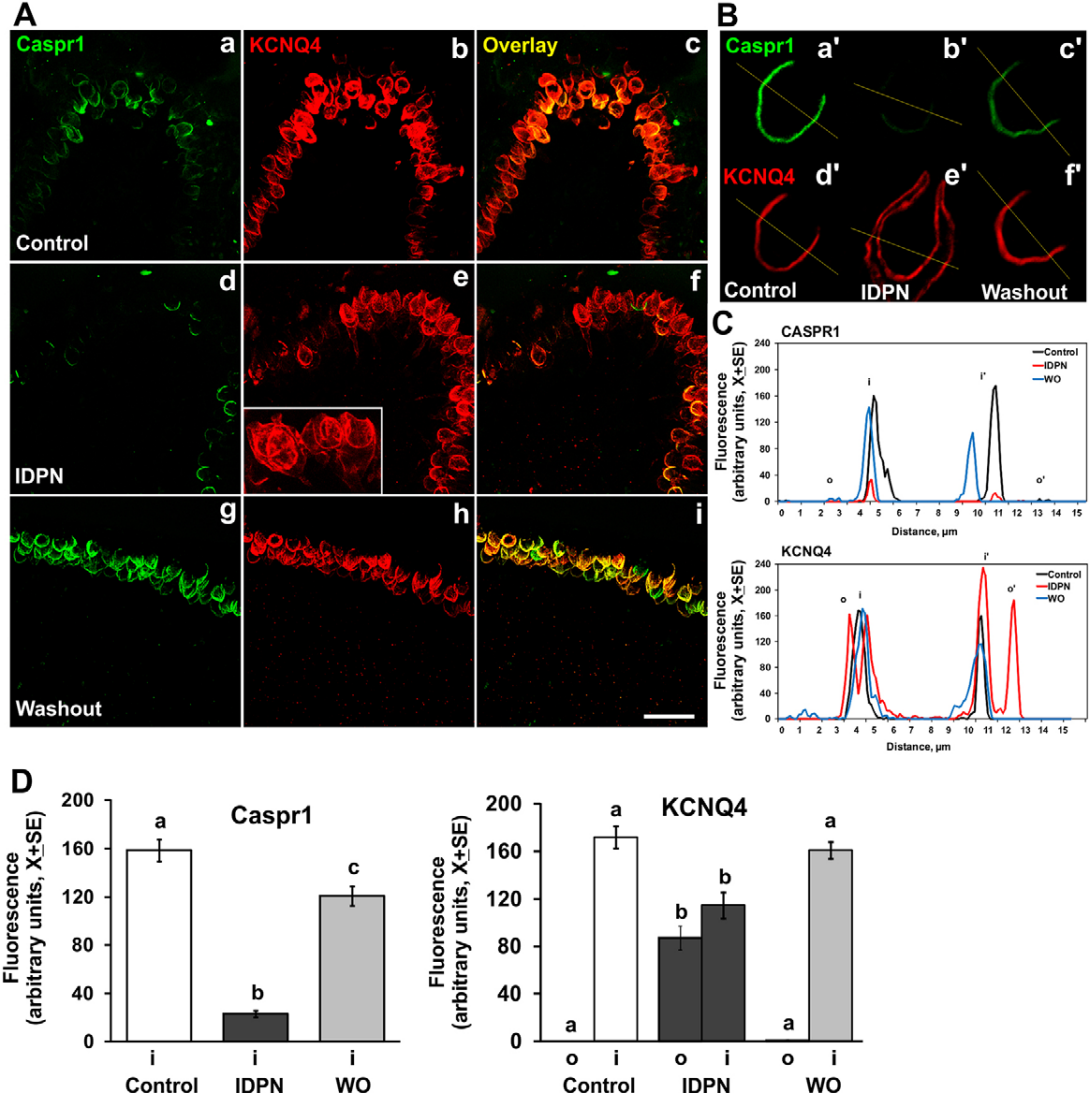
**Comparison of the effects of chronic ototoxic exposure (20 mM IDPN in drinking water for 4 weeks) and washout on the expression of CASPR1 and KCNQ4 in the vestibular sensory epithelium of the adult rat.** (A) Cristae immunolabeled with a guinea pig anti-CASPR1 antibody (green) and a rabbit anti-KCNQ4 antibody (red). Both labels show a similar distribution in control animals (a-c). After four weeks of exposure (d-f), CASPR1 immunofluorescence was much reduced, whereas KCNQ4 was redistributed within the calyx endings. The inset in e shows a higher magnification of the KCNQ4 label redistribution. A control-like distribution was observed after a 4-week washout period (g-i). Scale bar: 25 µm. (B) Higher magnification images of single calyx units. The same calices are shown in CASPR1 labeling (a′-c′) and KCNQ4 labeling (d′-f′). Immunofluorescence was quantified along 15 µm lines as depicted. (C) Representative profile plots obtained from the image analysis illustrated in B, from which peak fluorescence values for inner and outer faces of the calyces were obtained. (D) Mean group fluorescence values in the inner and outer faces of the calyceal membrane from control, treated and washout animals (*n*=30 cells from 3 animals per group). No significant CASPR1 immunolabeling was found on the outer faces in any experimental condition. a,b,c: groups not sharing a letter are significantly different, *P*<0.05, Duncan's test after significant ANOVA. WO, washout.

We also used immunofluorescence to examine the behavior of the voltage-gated potassium channel KCNQ4. We observed an abnormal distribution of this channel in calyceal contacts devoid of CASPR1 as a result of the 4-week 20 mM IDPN treatment (Fig. 5A,B). Thus, KCNQ4 immunoreactivity was similar on both the inner and outer faces of the calyceal endings after ototoxic exposure, in contrast to the distribution of the channel almost exclusively on the inner face of the control endings. Recovery of the normal distribution of KCNQ4 in the calyceal junction area was observed at the end of the washout period (Fig. 5B). For quantitative analysis, the intensity of fluorescence of CASPR1 and KCNQ4 was determined along 15 µm lines crossing the calyceal region, and the maximal values of the fluorescence peaks were determined for the inner and outer faces of each calyx. Representative images and graphical results are shown in Fig. 5B and 5C, respectively. Comparison of mean values (Fig. 5D) demonstrates the redistribution of the KCNQ4 immunofluorescence after IDPN exposure and recovery of the normal pattern at the end of the washout period (*F*[2,87]=11.02, *P*<0.001, and *F*[2,87]=76.09, *P*<0.001 for inner and outer faces, respectively) in parallel with the changes in CASPR1 content on the inner faces (*F*[2,87]=93.84, *P*<0.001) (in all cases, *n*=30 cells from three animals per group).

Taken together, these data demonstrate that during chronic IDPN ototoxicity the dismantlement of calyceal junctions is an early event preceding calyx detachment and fragmentation. This dismantlement includes a major loss of CASPR1 and tenascin-C, whereas other proteins characterizing this junction, although delocalized in their final placement, are present at normal levels at the terminal. The loss of CASPR1 and tenascin-C is not due to reduced gene expression in the neurons. Importantly, the data also demonstrate that rebuilding of the junctions occurs after termination of exposure to toxin.

### Disaggregation of the calyceal junctions occurs as individual events on a calyx-to-calyx basis

**Fig. 6.**
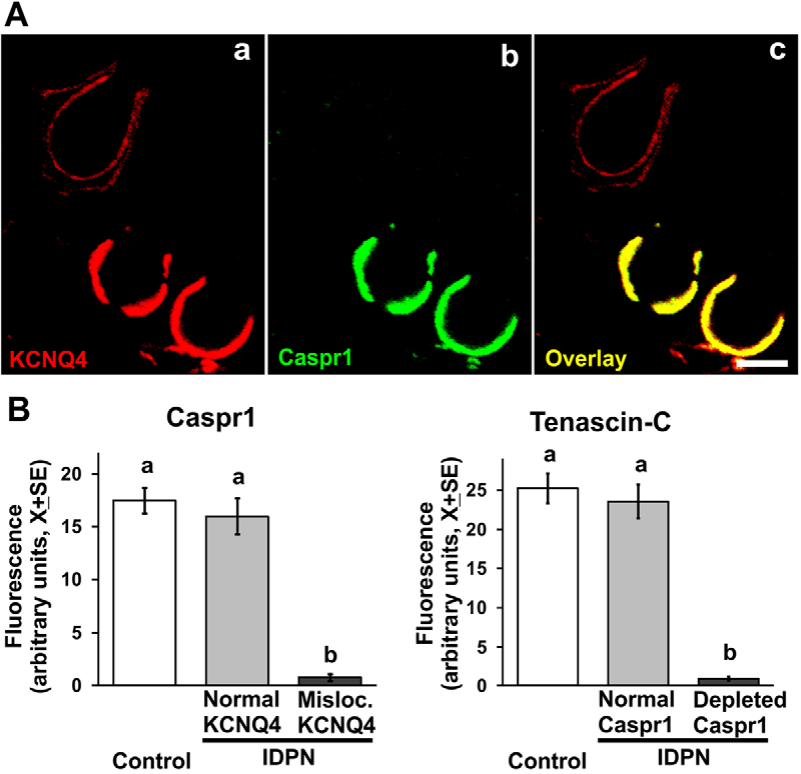
**Cell-to-cell basis of calyceal junction dismantlement.** (A) A vestibular epithelium from a rat exposed to 10 mM IDPN for 10 weeks. Note the two calyx units showing a normal distribution of KCNQ4 in the inner membrane of the calyx ending (bottom units), and the unit showing abnormal distribution (upper-left unit). CASPR1 immunofluorescence is normal in the first case and highly reduced in the second case (b,c). Scale bar: 5 µm. (B) Left: Quantitative analysis of CASPR1 fluorescence in normal and altered calyx units, as defined by their KCNQ4 distribution, demonstrating that unaffected calices from exposed animals have control-like CASPR1 content. Right: Quantitative analysis of tenascin-C fluorescence in normal and altered calyx units, as defined by their CASPR1 content, demonstrating that unaffected calices from exposed animals have control-like tenascin-C content. In both B and C, *n*=30 cells from 2-3 animals per group. a,b: groups not sharing a letter are significantly different, *P*<0.05, Duncan's test after significant ANOVA.

The previous observations demonstrated the loss and rebuilding of calyceal junctions, but did not reveal how that dismantlement takes place. One possibility would be that the neurons failed to maintain the calyceal junctions owing to the effect of IDPN on axonal transport. IDPN is known to impair axonal transport of neurofilaments (Griffin et al., 1978), although the mechanism involved is still unknown. To address these issues, we studied rats exposed to 10 mM IDPN for 10 weeks; the TEM images from our previous study (Seoane et al., 2001b) suggested that the dismantlement of the calyceal junctions would be ongoing in these animals. In these epithelia, individual calyceal units were observed either to display a control-like pattern, with colocalization of CASPR1, tenascin-C and KCNQ4 in the junction area, or to display an abnormal pattern, with markedly reduced levels of CASPR1 and tenascin-C and dispersed KCNQ4 labeling (Fig. 6A). The percentage of damaged calyces was very different in the three animals examined (0%, 6% and 63%) even though none of them showed overt behavioral evidence of vestibular dysfunction. However, most individual calyces could be classified as intact or damaged according to their normal or abnormal KCNQ4 localization. When calyces in these two groups were compared with each other and with calyces from control epithelia, CASPR1 immunoreactivity was found at control-like levels in intact calyces from intoxicated animals and severely depleted in damaged calyces from the same two animals. Thus, group differences in CASPR1 content (*F*[2,75]=32.80, *P*<0.001; Fig. 6B) were due to the reduced CASPR1 levels in cells defined by their abnormal KCNQ4 localization, but CASPR1 content was not reduced in other cells from the same treated animals. When the tenascin-C immunoreactivity was evaluated in calyceal units showing either intact or depleted CASPR1 content, a similar result was obtained: tenascin-C was found at control-like levels in intact calyces from intoxicated animals and severely depleted in calyces with depleted CASPR1 from the same two animals (*F*[2,72]=45.16, *P*<0.001; Fig. 6C). These data exclude the hypothesis that CASPR1 and/or tenascin-C are progressively lost during chronic toxicity owing to an inadequate supply of proteins caused by an effect of IDPN on their transport, because this effect is known to involve all axons and therefore parallel and progressive involvement of all terminals should occur. By contrast, the present data better fit the hypothesis that each single calyx is stable until a yet to be determined event triggers the dismantlement of the calyceal junction.

### Synaptic machinery is altered by chronic ototoxic exposure

**Fig. 7.**
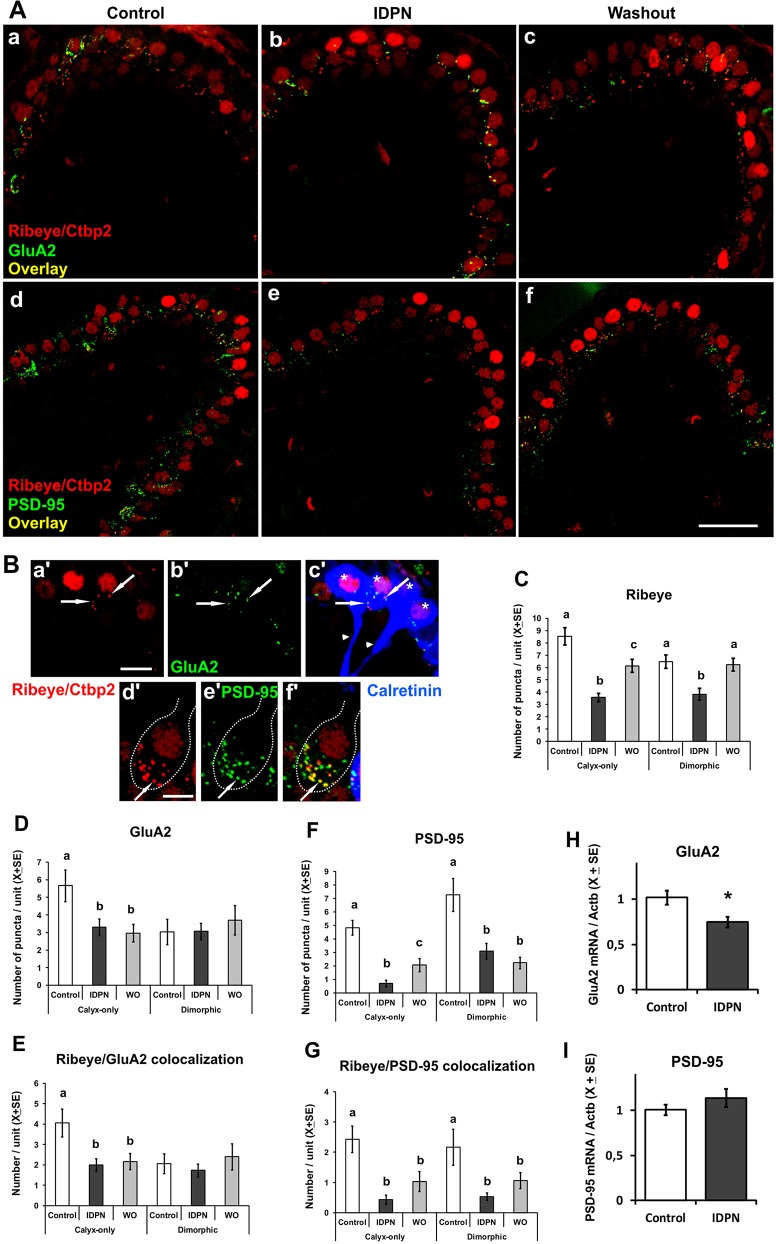
**Effects of chronic ototoxic exposure (20 mM IDPN in drinking water) and washout on synaptic proteins.** (A) Representative crista sections immunolabeled with antibodies against ribeye/Ctbp2 and GluA2 (upper row), and ribeye/Ctbp2 and PSD-95 (lower row), from control (a,d), exposed (b,e), and washout (c,f) animals. HC nuclei display Ctbp2 expression. At the level of the basolateral HC membranes, ribeye puncta identify pre-synaptic ribbons, whereas GluA2 and PSD-95 puncta characterize post-synaptic elements. Close proximity between the pre-synaptic and post-synaptic elements is revealed by either pairs of red and green puncta or a yellow colocalization label. Scale bar: 25 µm. (B) Examples of synaptic protein puncta in the two kinds of HCI/calyx units defined by calretinin labeling. Arrows indicate examples of matching puncta of ribeye and GluA2 in a HCI with a calyx-only afferent (a′,b′,c′), and of ribeye and PSD-95 in a HCI with a calyx from a dimorphic afferent (d′,e′,f′). In c′, calretinin label identifies four HCIs with calyx-only afferents (asterisks) arising from two terminals (arrowheads). In f′, the absence of calretinin label characterizes the dimorphic afferent. Scale bars: 10 µm in a′; 5 µm in d′. (C-G) Quantitative analysis of ribeye, GluA2 and PSD-95, as well as ribeye/GluA2 and ribeye/PSD-95 colocalizations by treatment group and HCI/calyx unit. Note the loss of synaptic puncta following ototoxic exposure, the stronger effect on PSD-95 compared with GluA2, the better recovery in ribeye than in post-synaptic puncta, and the differences between calyx-only and dimorphic units. Ribeye data (C) are from 60 cells from 6 animals per group, whereas GluA2, PSD-95 and colocalization data are from 30 cells from 3 animals per group. a,b,c: groups not sharing a letter are significantly different, *P*<0.05, Duncan's test after significant ANOVA. (H,I) RT-PCR of mRNA from vestibular ganglia displays reduced expression of *GluA2* but unchanged PSD-95 after IDPN exposure (*n*=7-8 per group, *P<0.05). WO, washout.

In the present study, we also evaluated the synaptic transmission machinery in the HCI-calyx synapses. Thus, we assessed the numbers of ribeye, GluA2 and PSD-95 immunoreactivity puncta, as well as the pairing of ribeye and GluA2, and of ribeye and PSD-95 puncta in control, 4-week-20 mM, and washout animals. The data obtained (Fig. 7) include fewer pre- and post-synaptic label pairs than the total numbers of either type of puncta, as previously described in this synapse (Sadeghi et al., 2014). Co-labeling with anti-calretinin antibodies allowed us to differentiate two types of calyx units: calyx-only (calretinin positive) or dimorphic (calretinin negative) afferents. Chronic IDPN toxicity caused a loss of ribeye puncta, with significant changes in group means in both calyx-only HCIs (*F*[2,177]=20.76, *P*<0.001) and dimorphic afferent HCIs (*F*[2,177]=7.92, *P*<0.001) (Fig. 7C; in each case *n*=60 cells from 6 animals per group). After the washout period, complete recovery was recorded in dimorphic afferents, but only partial recovery in calyx-only afferents. GluA2 (Fig. 7A,D; *n*=30 cells from 3 animals per group) was altered in calyx-only (*F*[2,87]=4.99, *P*=0.009) but not dimorphic (*F*[2,87]=0.291, not significant) afferents, and the observed effect persisted at the end of the 4-week washout period. The ribeye/GluA2 colocalization (Fig. 7E; *n*=30 cells from 3 animals per group) showed a similar pattern. A decreased number of pairs in calyx-only afferents was observed after treatment and persisted at the end of the washout period (*F*[2,87]=5.51, *P*=0.006), whereas no change in numbers was apparent in dimorphic afferents (*F*[2,87]=0.44, not significant).

Alterations in PSD-95 puncta numbers (Fig. 7F) were recorded for both calyx-only HCIs (*F*[2,87]=22.24, *P*<0.001) and dimorphic afferent HCIs (*F*[2,87]=10.83, *P*<0.001) (in each case *n*=30 cells from 3 animals per group). In both cases, significant decreases occurred after IDPN administration, and only the calyx-only HCI units showed a partial recovery in the washout animals; no recovery in PSD-95 puncta numbers occurred in dimorphic afferents. The numbers of ribeye/PSD-95 pairs showed significant differences for calyx-only HCIs (*F*[2,87]=9.76, *P*<0.001) and dimorphic afferent HCIs (*F*[2,87]=4.75, *P*=0.011) (in each case *n*=30 cells from 3 animals per group), which in all cases resulted from a significant drop in treated animals with no recovery after washout (Fig. 7G).

The mRNA expression levels of GluA2 in the vestibular ganglia were significantly reduced after IDPN exposure (Fig. 7H; *P*<0.05). Therefore, decreased gene expression may account, at least in part, for the observed decreases in GluA2 puncta. By contrast, no changes occurred in PSD-95 mRNA levels (Fig. 7I).

These data demonstrate that the alteration caused by chronic IDPN ototoxicity in the HCI-calyx relationship includes the synaptic machinery, and that repair to a control-like situation may not be achieved at a time point of recovery at which virtually complete repair of calyceal junctions has been observed.

## DISCUSSION

### The IDPN rat model for chronic vestibular toxicity and recovery

Previous research in this and other laboratories has demonstrated that IDPN and other nitriles are ototoxic compounds with many similarities with the aminoglycoside antibiotics (see Introduction). This study shows that drinking water exposure to IDPN induces vestibular dysfunction in a progressive manner, and that different degrees of functional recovery occur if exposure is halted at different time points. Therefore, this model may be useful to identify the cellular and molecular basis of vestibular dysfunction and recovery associated with chronic ototoxicity and washout. Although the literature contains a noticeable number of references on the CNS toxicity of IDPN (Wang et al., 2015), their value is dubious as they ignore the firmly established fact that the effects of IDPN on spontaneous motor behavior are a syndrome of vestibular dysfunction (Llorens et al., 1993; Llorens and Rodríguez-Farré, 1997; Boadas-Vaello et al., 2005; Soler-Martín et al., 2007; Saldaña-Ruíz et al., 2012a,b). The test battery used in the present study to assess vestibular dysfunction is highly specific and not affected by the alleged CNS effects of IDPN (Llorens and Rodríguez-Farré, 1997; Seoane et al., 1999; Boadas-Vaello et al., 2005).

### Loss of vestibular function precedes stereociliary damage and HC loss during chronic ototoxic exposure

Irreversible loss of vestibular function may result from HC degeneration caused by ototoxic drugs. However, HC degeneration may not be the only phenomenon involved. In this study, many animals with significant vestibular dysfunction showed a normal density of hair bundles with a control-like morphology, even though some of them displayed blebs. These blebs are known to be reversible and are often found in control specimens as they can appear within a few minutes of HC stress (Shi et al., 2005; Goodyear et al., 2008). A comparison of animals allowed or not allowed to recover from chronic IDPN ototoxicity indicated that functional recovery is possible if no significant HC loss or stereociliary coalescence has occurred. Longer intoxications are associated with more persistent dysfunction, greater HC loss and more extensive stereociliary damage. Differing from the reversible blebs, the loss of stereocilia individuality involves displacement of cytoplasmic organelles to the spaces created between the stereociliary actin filaments and this starts the extrusion of the HC from the epithelium (Li et al., 1995; Seoane et al., 2001a). Although repair of stereociliary bundles has been demonstrated in cultured bullfrog saccules and postnatal rat utricles (Gale et al., 2002; Zheng et al., 1999), the capacity for similar repair processes has not been established in the vestibular system of adult mammals *in vivo*, in which stereociliary coalescence may demarcate the point of no return towards HC demise.

### Dismantlement of calyceal junctions is an early event in chronic IDPN ototoxicity that is followed by calyx-ending fragmentation

Ultrastructural observations revealed a striking loss of calyceal junctions. Furthermore, confocal microscopy data showed major decreases in immunofluorescence labels for the junction components CASPR1 and tenascin-C. The specificity of the effect was supported by the KCNQ4 label, which did not decrease, but redistributed to a similar distribution between the inner and outer calyceal membranes. This abnormal distribution is similar to that described in *Caspr1*-null mice, which fail to generate calyceal junctions (Sousa et al., 2009). A hint as to how the dismantlement of the calyceal junctions occurs was obtained from epithelia from animals exposed for longer times at lower concentrations. These did not show partial reductions in CASPR1 and tenascin-C content in all calyx units, but a major reduction in some calyces and control-like levels in others. Therefore, the loss of calyceal junctions does not occur in parallel in all calyces, but seems to be triggered on a calyx-by-calyx basis. Importantly, this makes it unlikely that the present observations are due to the deleterious effects of IDPN on axonal transport (Griffin et al., 1978), which has been associated with depletion of neurofilaments in distal end zones, including vestibular afferents and motoneuron terminals (Seoane et al., 2003; Soler-Martín et al., 2012, 2014). Although the effect is highly specific for these cytoskeletal proteins, accumulations of neurofilaments in the vestibular ganglion perikarya and proximal axons (Llorens and Demêmes, 1996) and a reduction of neurofilament content in the afferents (Seoane et al., 2003) may eventually interfere with the supply of proteins required to maintain the calyceal junctions. However, if an inadequate supply of calyceal junction constituents was the cause of junction loss, one would expect a progressive reduction affecting all the calyces similarly, because IDPN affects transport similarly for all axons (Komiya et al., 1986).

Calyx endings were also observed to fragment, denuding the HCI basolateral membrane. This only occurred in animals presenting the worst behavioral condition, complete loss of calyceal junctions and initial stages of stereociliary coalescence; therefore, it may be concluded that calyx fragmentation is a later step that occurs after calyceal junction dismantlement. Also, the fragmented calyces were not overtly swollen, indicating that fragmentation was unlikely to be a consequence of swelling. The lack of overt calyx swelling contrasts with our previous observations with this model (Seoane et al., 2001b, 2003); the more rapid fixation procedure used in the present study, in which we used a fast immersion procedure instead of the intra-cardiac perfusion used in the previous studies, probably accounts for this difference. In relation to this point, it is worth mentioning the recorded reduction in *GluA2* mRNA expression after treatment. The absence of the GluA2 subunit in AMPA receptors determines their calcium permeability (Geiger et al., 1995; Lau and Tymianski, 2010), so it is possible that calyx endings from animals bearing a chronic ototoxic insult have increased susceptibility to excitotoxic damage, which is the probable cause of the calyx swelling (Brugeaud et al., 2007). Further studies are needed to address this intriguing possibility.

### Synaptic alterations in chronic IDPN ototoxicity

Chronic IDPN ototoxicity also altered the expression of the synaptic elements in the HCI-calyx units. The observed decrease in ribeye puncta probably implies reduced capacity of HCs for vesicular fusion and, thus, glutamate release (Zanazzi and Matthews, 2009). Reduced PSD-95 puncta can also be interpreted as denoting a reduction in synaptic strength (Zhang and Lisman, 2012). More discussion is necessary for the GluA2 immunoreactivity data. On the one hand, GluA2 puncta were reduced in the calyx-only but not in the dimorphic units, indicating that this change was not a direct consequence of calyceal junction loss, which affected all calyces similarly. On the other hand, these changes were associated with reduced mRNA expression; this is in contrast to the lack of alteration in PSD-95 mRNA expression. Downregulation of GluA2 has been demonstrated to occur in cultured neurons in response to inhibition of neuronal activity (Bai and Wong-Riley, 2003; Schonfeld-Dado et al., 2009; Orlandi et al., 2011). Therefore, the present data do not support our initial hypothesis (Sedó-Cabezón et al., 2014) that HCs under ototoxic stress release excessive glutamate and that consequent excitotoxicity causes the post-synaptic events. Conversely, they favor the hypothesis that chronic IDPN ototoxicity causes a reduction in neurotransmission in the calyces, and that compensatory responses are triggered at the gene expression level in the post-synaptic vestibular neuron.

### Afferent repair and persistence of synaptic alterations

Ultrastructural repair of auditory and vestibular afferents has been demonstrated in mammals after acute excitotoxic damage, in parallel with functional recovery (Puel et al., 1995; Brugeaud et al., 2007). Also, repair of vestibular afferents has been thoroughly characterized in pigeons after an intralabyrinthine exposure to streptomycin causing complete loss of HCs (Zakir and Dickman, 2006; Haque et al., 2009). In this model, HC regeneration is followed by afferent regeneration and the whole process associates with functional recovery. However, afferent growth progresses slowly and the final terminal morphologies are smaller and less complex than the original ones. On a related note, recent evidence indicates that permanent loss of auditory afferent terminals may result from exposure to noise levels just below those causing HC degeneration (Kujawa and Liberman, 2009), and that a loss of synaptic structures may be an early cause of an age-related decline in hearing function (Sergeyenko et al., 2013). The present data demonstrate the full capacity of calyx endings for calyceal junction rebuilding after termination of a chronic ototoxic insult. However, their capacity for reparation in the case of deeper lesions remains to be determined. We also found that alterations in post-synaptic proteins persisted at the end of the washout period. Therefore, the alterations in synaptic elements do not merely show secondary changes to the alterations in calyceal shape and calyceal junctions. Because of the known acute and chronic regulation of these elements in glutamatergic synapses, it seems likely that these persistent alterations would resolve after longer washout periods. Nevertheless, the possibility that they persist indefinitely as a ‘molecular scar’ of the ototoxic insult is clearly an open question.

### Relationship between behavioral deficits and afferent damage and repair

The recorded full behavioral recovery was roughly paralleled by repair of the afferents and the calyceal junctions, but without a full recovery of a control-like distribution of the synaptic proteins. Also, data from animals exposed to the lower concentration of IDPN indicate that calyceal junction dismantlement may precede the behavioral effects. This mismatch between the functional and molecular observations has several possible explanations that are not exclusive. First, our study concentrated on the calyx endings, so the changes in synapses between button endings and HCII in this model remain to be studied. Second, the recent discovery that glutamate spillover plays a significant role in neurotransmission at the calyx (Sadeghi et al., 2014) indicates that good transmission may occur even with a persistent pronounced effect on PSD-95 expression, in both dimorphic units showing recovered ribbon numbers and control-like GluA2 expression, and calyx-only units showing only partial recovery of ribbons and sustained reduced GluA2 content. Third, calyx units may constitute ‘high-frequency event’ detectors (Sadeghi et al., 2014), so the functional deficits associated with calyx dysfunction may require specific high-frequency testing and remain below the sensitivity threshold of the behavioral test battery used here. Fourth, other modifications not revealed by the present ultrastructural and immunohistochemical methods (e.g. changes in endolymph composition or mechanotransduction channel function) may be responsible, in part or chiefly, for the behavioral deficits observed.

### Relevance for ototoxicity in humans

Significant human exposure to IDPN does not occur. However, IDPN toxicity provides a dependable model that has many advantages over aminoglycosides for ototoxicity research in rats and mice. Several factors make it plausible that the present observations are relevant to humans exposed to aminoglycoside antibiotics. First, as discussed above, the main ototoxic properties of IDPN in rodents closely match those defined for the antibiotics (Llorens et al., 1993; Llorens and Demêmes, 1994; Crofton et al., 1994; Boadas-Vaello et al., 2005; Soler-Martín et al., 2007; Sedó-Cabezón et al., 2014). Second, the subsequent steps in HC demise following chronic IDPN, i.e. stereociliary fusion and HC extrusion (Seoane et al., 2001a; this study), have been well documented in aminoglycoside toxicity (Lenoir and Puel, 1987; Takumida et al., 1989; Li et al., 1995; Granados and Meza, 2005). Third, irrespective of the damaging agent, repair responses are intrinsic to the organism (Travo et al., 2012).

### Conclusion

This work describes afferent and synaptic damage and repair in adult mammals following chronic vestibular toxicity and subsequent recovery upon termination of exposure. The data demonstrate a robust capacity for calyceal junction rebuilding, and indicate that synaptic elements are differentially regulated across these processes. The observed cellular and molecular alterations are associated with, but do not totally account for, the vestibular dysfunction and recovery. Further studies with this model should reveal the mechanisms involved in dynamic maintenance of the calyx, calyceal junction and neurotransmission machinery, and the physiological relevance of these plasticity phenomena. In addition, the present data contribute to our understanding of clinical ototoxicity and open a new route to identification of targets for vestibular therapy. At present, options for treating vestibular disorders are practically inexistent.

## MATERIALS AND METHODS

### Animals and treatments

The care and use of animals were in accordance with Acts 5/1995 and 214/1997 of the Regional Government of Catalonia, and approved by the University of Barcelona's Ethics Committee on Animal Experiments. Eight- to nine-week-old male Long-Evans rats (CERJ, Le-Genest-Saint-Isle, France) were used. They were housed two or three per cage in standard Macrolon cages (215×465×145 mm) with wood shavings as bedding. They were acclimatized for at least 7 days before experimentation, and were maintained on a 12:12 h light:dark cycle (07:30-19:30 h) at 22±2°C and fed standard pellet diets (TEKLAD 2014, Harlan Laboratories, Sant Feliu de Codines, Spain) *ad libitum*. At regular intervals, the animals were weighed and evaluated for overall toxicity with regard to criteria for the ethical limits of suffering. According to this evaluation, only one animal out of 99 had to be eliminated from the study because it had lost >20% of its initial body weight.

Ninety-nine rats were exposed to 0 (*n*=29, controls), 10 (*n*=12) or 20 (*n*=58) mM IDPN (>98%, TCI Europe, Zwijndrecht, Belgium) in their drinking water. Water bottles were changed weekly and weighed to obtain rough estimates of the dose received by the animals. The 10 mM rats were exposed for 4 (*n*=4) or 10 (*n*=8) weeks, whereas the 20 mM rats were exposed for 4 (*n*=34), 6 (*n*=12) or 10 (*n*=12) weeks. The animals exposed for 4 weeks to 10 mM were killed at the end of the exposure period. Half of the other animals exposed for 4 or 10 weeks were killed at the end of the exposure period, whereas the remaining half were killed after a recovery period of the same length as the exposure period. The animals exposed for 6 weeks were killed at the end of the exposure (*n*=4), or after 6 (*n*=3) or 12 (*n*=4) weeks of recovery. Control animals were killed at the same time points for parallel processing of the samples. The animals were blindly examined for vestibular dysfunction before treatment and at weekly intervals during the exposure and recovery periods. To select animals for processing or survival, they were grouped into pairs (or trios) according to their vestibular dysfunction rating (VDR) at the end of the exposure period. From each pair (or trio) of animals showing a similar functional loss, one of them was processed at that time point, and the other(s) were monitored for functional recovery during the washout period and examined after the recovery period(s). This experimental approach served as a surrogate for repeated observation of the same animal.

For tissue collection, the rats were anesthetized with intraperitoneal injection of 400 mg kg^−1^ chloral hydrate and decapitated. In most cases, the temporal bones were directly immersed in cold fixative solution for immediate dissection of the vestibular sensory epithelia in a fume hood. The epithelia from the first ear were obtained less than 5 min after decapitation, and prepared for scanning (SEM) and transmission (TEM) electron microscopy. The epithelia from the second ear were obtained less than 15 min after decapitation and used for immunohistochemistry. In some experiments, vestibular epithelia were obtained for immunohistochemistry only. In one experiment, vestibular ganglia and epithelia from both ears were obtained for RT-PCR studies. In all cases, the samples were individually coded so that the different data pertaining to the same animals could be compared. Not all animals, treatment conditions, or time points were examined; the numbers of animals evaluated in each case are indicated throughout the text and provided in supplementary material Table S1 for further clarity.

### Vestibular dysfunction ratings (VDRs)

The alteration in vestibular function was determined using a test battery that has been proven to selectively assess vestibular dysfunction in rats (Llorens et al., 1993; Llorens and Rodríguez-Farré, 1997; Boadas-Vaello et al., 2005; Desmadryl et al., 2012). Briefly, the battery includes six measures that are rated 0 (normal behavior) to 4 (maximal deficit in behavior) from which a summary score (VDR, 0 to 24) is obtained by adding up the scores for all the behavior patterns. The battery includes three measures of spontaneous behavior (circling, retropulsion and abnormal head movements) that are characteristic of the waltzing phenotype that results from loss of vestibular function; and three vestibular reflexes: the tail-hang reflex, contact inhibition of the righting reflex and the air righting reflex tests. Further details have been published (Llorens et al., 1993; Boadas-Vaello et al., 2005).

### Electron microscopy

For electron microscopy, the vestibular epithelia were dissected in 2.5% glutaraldehyde in 0.1 M cacodylate buffer (pH 7.2). The samples were fixed overnight in the same solution, post-fixed for 1 h in 1% osmium tetroxide in cacodylate buffer, and dehydrated with increasing concentrations of ethanol, up to 100%. The utricle, the saccule and two cristae were used to examine surface preparations by SEM. To this end, the specimens were dried in a critical-point dryer using liquid CO_2_, coated with gold, and observed in a Quanta-200 SEM (FEI Company, Hillsboro, OR, USA). The third crista was embedded in Epon resin to be used for ultrastructural analysis by TEM. Semithin sections (1 µm) were stained with 1% Toluidine Blue and examined in a light microscope (Jenalumar, Zeiss, Jena, Germany). Ultrathin sections were stained with uranyl acetate and lead citrate and were observed with a JEOL 1010 microscope at 75-80 kV.

After the assessment of cristae and utriculi surfaces, animals were assigned a SEM Pathology Score according to the following scale: 0=no pathological features; 1=presence of few and small surface blebs behind the stereociliary bundles; 2=presence of larger and more abundant blebs; 3=presence of abundant blebs and very few (<5% of the population) damaged stereociliary bundles; 4=significant presence (5-40%) of damaged stereocilia bundles or missing HCs; 5=extensive presence (40-80% of the population) of damaged stereocilia bundles or missing HCs; 6=most HCs (>80%) are missing or show damaged stereocilia bundles.

### Antibodies and stains

The following antibodies were obtained as gifts from different researchers: guinea pig anti-CASPR1 (1:1000; from Manzoor Bhat, University of North Carolina; Sousa et al., 2009), rabbit anti-KCNQ4 (PB179 and PB180; 1:1000; Bechara Kachar, National Institute on Deafness and Other Communication Disorders, NIH; Beisel et al., 2005) and guinea pig anti-KCNQ4 (1:200; Thomas Jentsch, Leibniz-Institut für Molekulare Pharmakologie and Max-Delbrück-Centrum für Molekulare Medizin, Berlin, Germany; Spitzmaul et al., 2013). Mouse monoclonal anti-CASPR1 (clone K65/35, 1:400) and anti-PSD95 (clone K28/43; 1:200) antibodies were obtained from the UC Davis/NIH NeuroMab Facility. Other antibodies used were: rabbit anti-calretinin (7699/3H, Swant, Marly, Switzerland; 1:500), anti-myosin VIIa (25-6790, Proteus Biosciences, Ramona, CA, USA; 1:400), anti-neurofilament 200 (N4142, Sigma, Tres Cantos, Madrid, Spain; 1:1000) anti-tenascin (AB19013, Millipore, Madrid, Spain; 1:200), and the mouse monoclonals anti-GluA2 (clone 6C4, Millipore; 1:100), anti-neurofilament 200 (clone N52, Sigma; 1:1000) and anti-Ribeye/CtBP2 (clone 16/CtBP2, BD Transduction Labs; 1:100). Some of these primary antibodies were used as complementary markers but were not used to generate data included in the present report. In many cases, DRAQ5 (Abcam; 1:1000) was also used to label nuclei. For immunofluorescence labeling we used (1:500) Alexa Fluor 488-, 555- and 647-conjugated secondary antibodies (Invitrogen) against mouse (A21202, A21425, A31571), rabbit (A21206, A31572, A31573) or guinea pig (A11073) IgGs, and isotype-specific against mouse IgG1 and IgG2a (A21131, A21127). Data included in this article were obtained with primary antibodies for which specificity has been well established, as shown in supplementary material Table S2. In addition, we confirmed the lack of non-specific staining when the primary antibody was omitted. Non-specific staining was found only in blood vessels for some secondary antibodies, but these were not used to obtain the data presented here.

### Immunohistochemistry

The vestibular epithelia were dissected and fixed for 1 h at room temperature in 4% paraformaldehyde in PBS, and stored at −20°C in a cryoprotective solution (34.5% glycerol, 30% ethylene glycol, 20% PBS, 15.5% distilled water). Immunohistochemistry analysis was performed on whole vestibular epithelia. To eliminate possible bias due to day-to-day differences in staining outcomes, equal numbers of epithelia from each experimental group were processed in parallel in all runs of the immunolabeling protocol. Tissues were first incubated for 90 min in 4% Triton X-100 and 20% donkey serum in PBS. Primary antibodies were incubated in 0.1% Triton X-100 and 1% donkey serum in PBS for 48 h at 4°C. Secondary antibodies were incubated in 0.1% Triton X-100 in PBS overnight at 4°C. Specimens were thoroughly rinsed with PBS after each incubation period. Incubation with DRAQ5 was performed in PBS for 15 min between the final washes. After immunostaining, the epithelia were embedded in 0.49% gelatin, 30% bovine serum albumin and 20% saccharose in PBS overnight at 4°C. The epithelia were then oriented in the plane formed between two successively formed blocks of the same media solidified with 9% glutaraldehyde. The specimens were then sectioned at 40 µm in a Leica VT100M vibrating microtome, and the sections mounted in Mowiol medium.

### Confocal microscopy and image analysis

Sections were observed using a Leica TCS-SL confocal microscope. Each result included in this report was obtained from a minimum of three crista from each of three different animals. The whole crista was sectioned and all sections were examined. For quantitative analysis, images were obtained from optimally oriented sections with the 63× objective at zoom level 2. The same image acquisition settings were used with the samples from the different groups of animals analyzed. *Z*-stacks of optical sections 0.5 µm thick were obtained spanning 25 µm. These were combined into 3- or 5-µm thick images by serial projection of six or ten consecutive images into snapshots that were used for quantitative analysis. To compare the intensity of the antibody labeling, arbitrary fluorescence units were obtained using the scan ROI tool of the Leica LCS Lite program, with a single predefined 100 µm^2^ region of interest. To compare protein distribution across calyx membranes, the profile of fluorescence intensity across a line was obtained using ImageJ software (National Institute of Mental Health, Bethesda, Maryland, USA). To characterize synaptic elements, the number of Ribeye/CtBP2 puncta, GluA2 puncta and PSD-95 puncta, as well as the Ribeye-GluA2 and Ribeye-PSD-95 colocalization, were determined in two different HCI-calyx units: calyx-only (identified by calretinin labeling of the calyx afferent) and dimorphic afferents (with no calretinin label), both of them differing from HCIIs (identified by calretinin labeling of the HC). These quantitative data were obtained from at least three animals and at least ten cells per animal, distributed uniformly across a minimum of three representative sections per animal.

### RT-PCR

Ears were dissected in PBS and two tissue pools were obtained from each animal. One consisted of the two vestibular ganglia, and the other consisted of five cristae and two utricles; the sixth crista was processed for immunohistochemistry. The tissues were placed in RNAlater (Ambion) immediately after dissection. Total RNA was extracted the same day using the Illustra RNAspin Mini RNA Isolation Kit (GE Healthcare). After off-column cleaning with the DNase (GE Healthcare), equal quantities of total RNA (as estimated using a NanoDrop spectrophotometer; Wilmington, DE, USA) were reverse-transcribed using a High-Capacity cDNA Reverse Transcription Kit (Applied Biosystems). Equal amounts of cDNA (15 ng) were used for the RT PCR analysis. Actin beta (*Actb*) was selected as the internal control gene based on a preliminary study in which this gene was identified as the most stable of four reference genes (actin beta, glyceraldehyde-3-phosphate dehydrogenase, 45S pre-ribosomal RNA and TATA box binding protein) under our experimental conditions, as evaluated using Expression Suite software (Applied Biosystems). The RT-PCR was performed with a SensiFAST Probe Hi-ROX Kit (Bioline, Barcelona, Spain) and the following TaqMan assays (Life Technologies): Rn00667869_m1 (actin beta), Rn00568514_m1 (GluA2), Rn00583206_m1 (*Caspr1*), Rn00589080_m1 (contactin-1), Rn01454947_m1 (tenascin-C), Rn00583547_m1 (GluR3) and Rn00588099_m1 (PSD-95). Plates with 384 wells each containing 10 µl of reaction mixture were first incubated at 50°C for 2 min and then at 95°C for another 10 min, followed by 40 cycles (95°C for 10 s and then 60°C for 1 min) of PCR. All the samples were run in duplicate, and real-time fluorescence was detected using a 7900 HT Real-Time PCR System (Applied Biosystems). Threshold cycles (C_t_) were analyzed using Expression Suite software, and a relative quantification method (ΔΔC_t_) was used to calculate target gene expression according to the Guide to Performing Relative Quantitation of Gene Expression Using Real-Time Quantitative PCR (Applied Biosystems).

### Data analysis

Data are presented as mean±s.e.m., except where otherwise indicated. When possible, vestibular dysfunction data were analyzed with repeated-measures MANOVA (Wilks' criterion) with the day as the within-subject factor. The behavioral data from the 20-week experiment were not suitable for MANOVA analysis and were therefore analyzed using a repeated measures ANOVA design adjusted according to the Greenhouse-Geiser solution. Day-by-day analysis was performed after significant day-by-treatment interactions were recorded. Other data were analyzed by appropriate ANOVA designs and Duncan's post-hoc test, or by Student's *t*-test. The IBM SPSS Statistics 20 program package was used.
