# Transient alteration of the vestibular calyceal junction and synapse in response to chronic ototoxic insult in rats

**DOI:** 10.1242/dmm.026278

**Published:** 2016-07-01

**Authors:** Lara Sedó-Cabezón, Paulina Jedynak, Pere Boadas-Vaello, Jordi Llorens

There was an error published in *Dis. Model. Mech*. **8**, 1323-1337.

An erroneous TaqMan assay was used to evaluate the expression of the *PSD-95* gene by RT-PCR. Therefore, the data shown in Fig. 7I do not represent the expression levels of the *PSD-95* mRNA. This error has null impact on the main conclusions of the study.

The authors apologise to the readers for this error.

